# Denosumab versus zoledronic acid for preventing symptomatic skeletal events in Asian postmenopausal women with oestrogen-receptor-positive advanced breast cancer: an outcome analyses with a mean follow-up of 3 years

**DOI:** 10.1186/s12891-018-2338-6

**Published:** 2018-11-30

**Authors:** Chi Zhang, Fan Zhang, Guanzhao Liang, Xianshang Zeng, Weiguang Yu, Zhidao Jiang, Jie Ma, Mingdong Zhao, Min Xiong, Keke Gui, Fenglai Yuan, Weiping Ji

**Affiliations:** 1 Department of Joint surgery; Translational Research Centre of Regenerative Medicine and 3D Printing Technologies of Guangzhou Medical University, The Third Affiliated Hospital of Guangzhou Medical University, Duobao Road No.63, Liwan District, Guangzhou, 510150 Guangdong China; 2 Department of Radiology, The First Affiliated Hospital of Sun Yat-sen University, Huangpu East Road No. 183, Huangpu District, Guangzhou, 510700 Guangdong China; 3 Department of Emergency, The First Affiliated Hospital of Sun Yat-sen University, Huangpu East Road No.183, Huangpu District, Guangzhou, 510700 Guangdong China; 4 Department of Orthopaedics, The First Affiliated Hospital of Sun Yat-sen University, Huangpu East Road No. 183, Huangpu District, Guangzhou, 510700 Guangdong China; 5 Department of breast surgery, Hongkong Elizabeth hospital, Gascoigne Road No.30, Kowloon, Hongkong, Kowloon, China; 6 Department of Pharmacy, The First Hospital of Jilin University, Changchun, Jilin, 130021 China; 7 Department of Orthopaedics, Jinshan Hospital, Fudan University, Longhang Road No. 1508, Jinshan District, Shanghai, 201508 China; 8 Department of Orthopaedics and Central Laboratory, The Third Hospital Affiliated to Nantong University, Wuxi, 214041 Jiangsu China; 9 Department of General Surgery, The second affiliated hospital and Yuying children’s hospital of Wenzhou Medical University, Zhejiang, 325003 China

**Keywords:** Denosumab, Zoledronic acid, Breast cancer, Symptomatic skeletal event, Outcome analyses

## Abstract

**Background:**

The purpose of this study was to evaluate the efficacy of denosumab or zoledronic acid (ZA) using symptomatic skeletal events (SSEs) as the primary endpoint in Asian postmenopausal women with oestrogen-receptor-positive advanced breast cancer.

**Methods:**

Asian postmenopausal women with oestrogen-receptor-positive advanced breast cancer receiving subcutaneous denosumab 120 mg Q4W, or intravenous ZA 4 mg Q4W until the primary analysis cut-off date were retrospectively analysed in the Hong Kong Practice-Based Cancer Research Center(HKCRC) from March 2011 to March 2013. The time to first on-study SSE that was assessed either clinically or through routine radiographic scans was the primary endpoint.

**Results:**

242 patients received denosumab or ZA treatment (*n* = 120, mean age of 64.9 years (SD 3.01) and *n* = 122, 65.4 years (3.44), respectively). The median times to first on-study SSE were 14.7 months (12.9–45.6) and 11.7 months (9.9–45.6) for denosumab and ZA, respectively (hazard ratio, HR 0.44, 95% CI 0.71–2.95; *p* = 0·0002). Compared with the ZA group, denosumab-treated patients had a significantly delayed time to first SSE (HR 0.65 [95% CI 0.29–1.45], *p* < 0.0001). An increased incidence of SSE was found in the 16-month follow-up with rates of 2.1 and 10.7% for denosumab and ZA, respectively (*P* = 0.033). The difference persisted with time with rates of 8.3 and 17.2% at the final follow-up, respectively (*P* < 0.05).

**Conclusion:**

In postmenopausal women aged ≥60 years with oestrogen-receptor-positive advanced breast cancer, denosumab significantly reduced the risk of developing SSEs compared with ZA. The findings of this pilot trial justify a larger study to determine whether the result is more generally applicable to a broader population.

## Background

Oestrogen-receptor-positive advanced breast cancer, one of the most common malignancies reported worldwide, has a protracted risk of symptomatic skeletal events(SSEs), which are defined as radiation to the bone, symptomatic pathologic fracture, surgery to the bone, or symptomatic spinal cord compression [1–4]. SSEs often occur in women with oestrogen-receptor-positive advanced breast cancer and the associated complications have a substantial disease and economic burden. After 5 years of adjuvant tamoxifen, patients have a sustained risk of SSEs [5]. Long-term follow-up from pivotal upfront trials of adjuvant aromatase inhibitors, including Arimidex and Tamoxifen, alone or in combination, have demonstrated a continuing rate of SSEs of approximately 11% per year after initial therapy [6, 7]. These findings emphasize the need for extended adjuvant therapy.

Denosumab, a fully human monoclonal antibody, neutralizes the Receptor Activator of Nuclear Factor Kappa-B Ligand (RANKL), which is essential for the formation, function, and survival of the osteoclasts; also, it blocks osteoclast-mediated bone resorption [8, 9]. Gnant et al. [1] described the application of denosumab for preventing SSEs in postmenopausal women with oestrogen-receptor-positive stage 1–3 breast cancer undergoing denosumab as adjuvant treatment. Nevertheless, their study subjects who had a bone mineral density (BMD) T-score < − 2.5 at study entry failed to be specifically excluded. Furthermore, other risk factors influencing the occurrence of SSEs in their study subjects were not described. The ZO-FAST study [10] of zoledronic acid (ZA), which was also performed in postmenopausal women with oestrogen-receptor-positive stage 1–3 breast cancer every 6 months, revealed no difference in the SSE rates in the long-term follow-up. In another AZURE trial [11] evaluating ZA, the time to first SSE was reduced with the exploitation of ZA; however, this difference was attributable to the effect on SSEs after breast cancer recurrence, and there was no difference in the SSE rates before breast cancer recurrence.

For Asian postmenopausal women with oestrogen-receptor-positive advanced breast cancer, to the best of our knowledge, a direct comparison between denosumab and ZA has rarely been reported in the published literature. Denosumab and ZA are effective interventions to prevent SSEs, and they provide a new bone protection option for postmenopausal women with oestrogen-receptor-positive advanced breast cancer. Nevertheless, several unanswered questions remain [12]. The purpose of the retrospective study was to evaluate the efficacy of denosumab or ZA using SSEs as the primary endpoint in Asian postmenopausal women with oestrogen-receptor-positive advanced breast cancer.

## Methods

### Study population

Subjects included in this analysis were collected from the Hong Kong Practice-Based Cancer Research Center (HKCRC). Eligible patients were Asian postmenopausal women aged ≥60 years with histologically or cytologically confirmed oestrogen-receptor-positive advanced breast cancer diagnosed from March 2011 to March 2013. All patients had completed treatment with surgery and/or radiation and chemotherapy ≥4 weeks before study enrolment and received adjuvant denosumab or ZA therapy. The main exclusion criteria were the following: previous denosumab or ZA exposure or any aromatase inhibitor use during the 3 months; non-healed or planned surgery; chemotherapy or endocrine therapy for other diseases; active metabolic bone disease; undergoing castration; recurrence and metastasis before SSEs; use of medication that affects bone metabolism; renal deterioration during treatment; other primary tumours or advanced cancer; discontinuation or interruption of denosumab or ZA treatment; unable to complete surveys; life expectancy < 1 year; a BMD T score ≤ − 2.5 SD at the lumbar spine or femoral neck; modification of therapy during the follow-up period; severe infectious diseases; an American Society of Anesthesiologists (ASA) score of IV or V; and co-occurring mental illness.

### Definitions of the descriptive variables

The patients’ body mass index (BMI) was calculated during the clinical examination. The BMD T-score was measured at the lumbar spine or femoral neck. Radiographic skeletal surveys of SSEs confirmed radiologically by a radiologist were performed within 4 weeks, every 3 months thereafter, and at the end-of-study visit. SSEs were defined as radiation therapy to the bone (including radioisotopes), pathologic fracture (excluding trauma), surgery to the bone, or spinal cord compression.

### Study design and treatment

This was a retrospective study in which postmenopausal women with oestrogen-receptor-positive breast cancer underwent either subcutaneous denosumab 120 mg Q4W or intravenous ZA 4 mg Q4W until the primary analysis cut-off date. The denosumab and ZA doses were not adjusted or suspended. All patients were instructed to take calcium (1 g/day) and vitamin D (400 IU/day). The same antineoplastic therapy was routinely used and was not adjusted in the two groups.

### Statistical analysis

The descriptive and outcome variables were assessed by calculating the frequencies, means, and standard deviations (SDs). Comparison between groups was conducted using Pearson’s Chi-square test or the Mann–Whitney U test for continuous variables. The time to first SSE was assessed using a Kaplan-Meyer analysis and Log-rank test. The risk and factors of SSEs were analysed by the Cox regression model. The analyses were based on the data up to the primary analysis cut-off. A *p*-value < 0.05 was used as a threshold for significance. All analyses were conducted using SPSS software (IBM-SPSS Statistics version 23.0, Inc., New York, USA).

## Results

### Patients

**Fig. 1:**
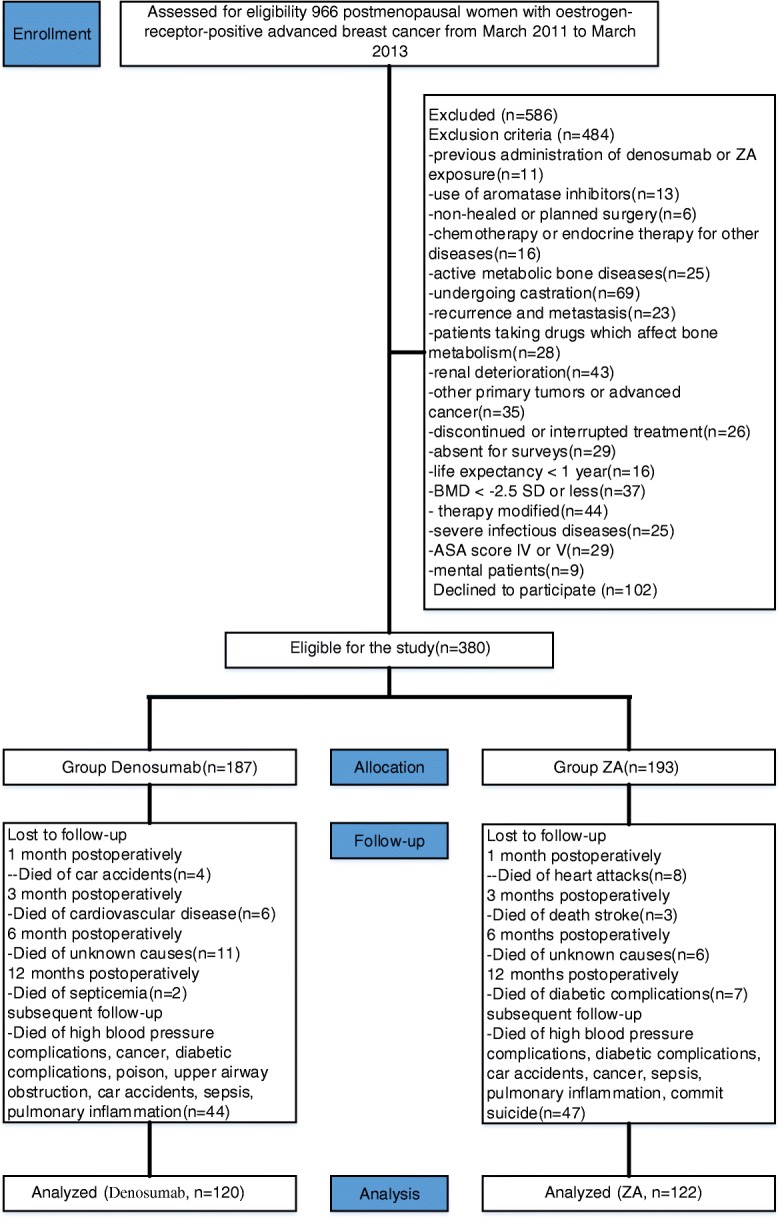
Flow diagram demonstrating methods for identification of studies to assess the efficacy of denosumab or zoledronic acid (ZA) using symptomatic skeletal events(SSEs) as the primary end point in postmenopausal women with oestrogen-receptor-positive advanced breast cancer

A total of 966 patients were assessed for study eligibility. Of these, 242 patients (denosumab group *n* = 120, mean age 64.9 years (SD 3.01) and ZA group *n* = 122, 65.4 years (SD 3.44), respectively) met the inclusion criteria. Details are shown in Fig. 1 and Table 1. The median duration for study at the primary analysis cut-off date was 37 months (IQR 25.1–45.3) for patients on denosumab and 38 months (IQR 26.2–45.8) for those on ZA. The median time to first on-study SSE was 16.5 months (95% confidence interval [CI] 14.3–45.3) with denosumab compared to 11.7 months (9.9–45.6) with ZA (hazard ratio [HR] 0.44, 95% CI 0.71–2.95; *p* = 0.0002). Compared with the ZA group, denosumab-treated patients had a significantly delayed time to first SSE (HR 0.65 [95% CI 0.29–1.45], *p* < 0.0001). An increased incidence of SSE was found at the 16-month follow-up with rates of 2.1 and 10.7% for denosumab and ZA, respectively (*P* = 0.033). The difference persisted with time with rates of 8.3 and 17.2% at the final follow-up (*P* < 0.05).

**Table 1:** Comparison of patient demographics between groups

Variable	Denosumab (*n* = 120)	ZA (*n* = 122)	*P* – value
Age (years)	64.9 ± 3.01	65.4 ± 3.44	0.19^*a^
Menopause age	53.1 ± 1.82	52.9 ± 1.78	0.43^*a^
Pathological types, No.			0.92^*b^
Invasive ductal carcinoma	97	98	
Other pathological types	23	24	
Tumor staging			0.95^*c^
I	53	57	
II	46	40	
III	21	25	
BMI (kg/m^2^)	24.4 ± 2.34	23.9 ± 2.36	0.11^*a^
BMD	−2.30 ± 0.21	−2.33 ± 0.29	0.37^*a^
Personal history of fractures	15/120	19/122	0.49^*b^
Family history of fractures	17/120	18/122	0.90^*b^

^*^ *No statistically significant values.* ^*a*^ *Analysed using an Independent-Samples t-test;* ^*b*^ *Analysed using the Chi-square test;* ^*c*^ *Analysed using the Mann-Whitney test. ZA: zoledronic acid; SSEs: symptomatic skeletal events; BMI: body mass index; BMD: bone mineral density*

### The homogeneity test of variances and t test of clinical data

In this study, both the pathological type and tumour stage in the 2 groups of patients were similar. There were no significant differences in the clinical risk factors, including the age at diagnosis, age at menopause, BMI, personal or family fracture history, or chemotherapy history.

### SSE incidence and risk

In a comparison of the SSEs as endpoints, there were 11 fewer first on-study SSEs in the denosumab group than in the ZA group (10 versus 21, respectively) (Table 2). As anticipated, there were fewer first on-study pathologic fractures in the denosumab group than in the ZA group (4 versus 13). In addition to pathological fractures, the numbers of patients with confirmed first on-study SSEs in each remaining event type was lower in the denosumab group than in the ZA group.

**Table 2:** Comparison of SSE incidence between groups

Variable	Denosumab (*n* = 120)	ZA(*n* = 122)	*P* - value
Total incidence of SSEs	10/120	21/122	0.04^*a^
SSE incidence			
within 2 years	3/120	4/122	0.21^a^
after 2 years	7/120	17/122	0.04^*a^

^*^ *Statistically significant values. aAnalysed using the Chi-square test. ZA: zoledronic acid; SSEs: symptomatic skeletal events*

In the ZA group, the first on-study SSE incidence was 17.2% (21 /122), in which the first on-study SSE incidence during 2-year drug use was 3.3% (4/122). After 2-year treatment, 13.9% (17/122) of cases developed a first on-study SSE. In contrast, in the denosumab group, the incidence of first on-study SSEs was only 8.3% (10/120), which was lower than in the ZA group (*P* = 0.04). The incidence of first on-study SSEs was 5.8% (7/120) after 2 years, which was also significantly lower than that in the ZA group (P = 0.04), but there was no significant difference between groups in the first on-study SSE within the 2-year period [2.5% (3/120) vs. 3.3% (4/122), respectively, *P* = 0.21].

Compared with ZA, denosumab reduced the risk of first on-study SSEs by 20% (HR, 0.80; 95% CI 0.52–0.88; *P* = 0.002). The median time to the first on-study SSE was not reached in the denosumab group, while it was 25.5 months in the ZA group. As previously reported, denosumab reduced the risk of first on-study SSEs by 18% compared to ZA. The risk of a first on-study SSE in the ZA group was 10 times higher than that in the denosumab group during the 2-year drug use (HR = 10.843, *P* = 0.026). After 2-year treatment, the ZA group had an increased risk of a first on-study SSE compared with the denosumab group (HR = 13.221, *P* = 0.001).

### Cumulative risk of SSEs

**Fig. 2:**
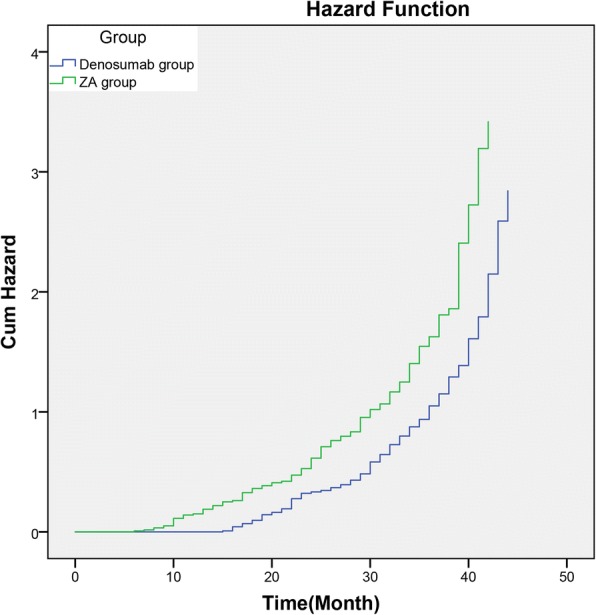
Kaplan-Meier curves of hazard

**Fig. 3:**
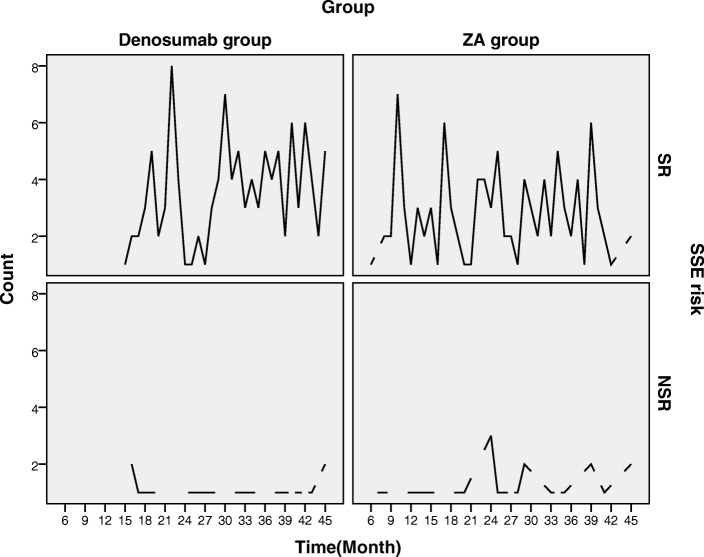
Comparison of the distribution and size of SR and NSR at each follow-up time point. There were statistically significant differences in SR or NSR between groups noted. SR: symptomatic skeletal event; NSR: non-symptomatic skeletal event

The Kaplan-Meyer curve showed that the cumulative risk curve in the denosumab group was significantly lower than that in the ZA group (P = 0.002) (Figs. 2, 3 and Table 3).

**Table 3:** SSE risk ratio between groups

Variable	Denosumab (*n* = 120)	ZA(*n* = 122)	*P* - value
Total SSE HR(95%CI)	12.43	19.92	0.002^*^
	(2.17-17.44)	(3.34–21.35)	
SSE HR(95%CI)			
within 2 years	6.33	7.27	0.106^*^
	(1.36-12.38)	(1.54–19.63)	
after 2 years	36.26	83.52	0.001^*^
	(3.31-161.15)	(4.29–92.60)	

^*^ *Statistically significant values. SSEs: symptomatic skeletal events; ZA: zoledronic acid; HR: hazard ratio; CI: confidence interval*

### Clinical risk factors for SSEs

In the denosumab group, the risk of a first on-study SSE in a person with a history of SSEs was approximately 15 times higher than for someone with no history of SSEs (HR =15.41, P = 0.001). The risk of a first on-study SSE with a family history of SSEs was approximately 2 times higher than for someone with no family history of SSEs (HR = 2.07, P = 0.001). The risk of a first on-study SSE in a denosumab-treated patient aged > 60 years was approximately 1-fold higher than in a ZA-treated patient (HR =1.59, P = 0.001). The clinical risk of other factors that influence the occurrence of SSEs, including menopausal patients aged ≤50 years and low BMI (≤ 19 kg /m^2^), increased the trend in the first on-study SSE risk, but no significant differences were observed (*P* > 0.05) (Table 4).

**Table 4:** Clinical risk factors for SSEs in two groups

	Denosumab		ZA	
Risk factors	HR(95%CI)	*P* –value	HR(95%CI)	*P* - value
Age (y)		0.003^*^		0.001^*^
≤60	1		1	
> 60	3.13(1.02–1.68)		4.72(2.36–7.17)	
Menopause age(y)		0.440		0.247
≤50	5.04(0.03–22.12)		5.04(0.03–22.12)	
> 50	1		1	
PHF		0.002^*^		0.005^*^
no	1		1	
yes	21.34(5.23–49.12)		36.75(2.39–74.32)	
FHF		0.037^*^		0.041^*^
no	1		1	
yes	6.79(1.10–11.65)		8.86(1.43–38.15)	
BMI(kg/m2)		0.793		0.219
≤19	3.76(0.66–31.32)		5.36(0.19–44.61)	
> 19	1		1	

^*^ *Statistically significant values. SSEs: symptomatic skeletal events; ZA: zoledronic acid; PHF: personal history of fractures; FHF: family history of fractures; BMI: body mass index*

## Discussion

In the retrospective study on 242 Asian postmenopausal women with oestrogen-receptor-positive breast cancer who underwent denosumab or ZA treatment, the most important finding was that denosumab-treated patients had a decreased risk of SSEs compared with ZA-treated patients, which coincided with the results of some other reports [1, 4, 21]. No safety differences were found in women treated with denosumab or ZA.

There was a recent review of the treatment and prevention of SSEs in postmenopausal women with oestrogen-receptor-positive breast cancer [9, 13, 14]. Previous therapy with aromatase inhibitors (AI), which reduce endogenous serum oestrogen concentrations and are related to accelerated bone loss, decreased the BMD and increased the SSE risk, making them important in the comprehensive treatment for women with oestrogen-receptor-positive breast cancer [14, 15]. Because AI can effectively reduce the levels of oestrogen in postmenopausal women with breast cancer, significantly reduce breast cancer recurrence and metastasis, and prolong patient survival, they have become one of the preferred endocrine therapy drugs for postmenopausal women with oestrogen-receptor-positive breast cancer [12, 16]. However, postmenopausal women, because the skeletal system has lost the protective effect of oestrogen, are at a high risk of SSEs, and AI will add an additional 3 to 4% / year bone loss, increasing the risk of SSEs [4]. In recent years, international scholars have focused their attention on a new drug (denosumab) and have comprehensively evaluated its effectiveness and safety [17, 18]. However, the findings in postmenopausal women have remained unclear and have demonstrated the need for further study [17–19]. Moreover, there are no literature data on the SSEs evaluated in Asian postmenopausal women.

There are many large-scale clinical trials studying the effects of denosumab in postmenopausal women with oestrogen-receptor-positive breast cancer [20–22], but the tests in the subgroup analysis of SSEs are limited because the control group is women receiving tamoxifen (TAM) [19]. Previous studies have shown that TAM has a weak oestrogen effect, which can protect the bone system in postmenopausal breast cancer women [23]. This interference factor may increase the risk of SSEs caused by denosumab treatment. In the current study, we selected postmenopausal women with oestrogen-receptor-positive breast cancer who received ZA as a control group to avoid the interference of endocrine therapy and accurately analyse the risk of denosumab-related SSEs. Statistical analysis showed that the clinical data of the patients in both groups failed to have selective bias. Furthermore, our analyses of SSEs excluded asymptomatic fractures and/or spinal cord compression. Because of any SSE having the potential to convert symptomatic over time, the number of SSEs could be underestimated in some cases [18].

Few previous reports have focused on denosumab versus ZA for preventing SSEs in Asian postmenopausal women with oestrogen-receptor-positive breast cancer in the Asian population [24–26]. However, the majority of these studies had low sample sizes and a relatively short follow-up period; therefore, drawing conclusions about the relative superiority of one drug over the other using SSEs as the primary endpoint may be inappropriate. Lipton et al. [27] reported that in breast cancer patients with bone metastases, denosumab was superior to ZA in delaying the time to the first on-study SSE (HR = 0.82; *P* = 0.01) and the time to the first and subsequent on-study SSEs (HR = 0.77; *P* = 0.001). Martin et al. [28] evaluated further results from this study (denosumab was shown to be superior to ZA in preventing SSEs in women with breast cancer in a randomized, double-blind phase III study) related to SSEs and showed that denosumab had superior efficacy in the time to first SSE compared with ZA. In this study, baseline characteristics of our population were consistent with a previously published study [21, 22], and the SSE rate in the denosumab group was significantly lower compared with that in the ZA group regardless of whether analysis was performed during drug use, after the cessation of drug use, or during the follow-up period. The total SSE incidence rates in the denosumab and ZA groups were 8.3 and 17.2%, respectively, which were lower than the 19.5% (40-month follow-up) and 24.8% (70-month follow-up) in the BIG1–98 trial. These differences may be related to the differences between the Chinese and Western race, physical quality and lifestyle. In addition, in the study, 2 (2/120) cases treated with denosumab for 2 years, who were followed up individually, had SSEs at the end of 9 and 16 months after treatment, respectively. The SSE incidence of denosumab-treated patients (8.3%, 10/120) was significantly lower than the 17.2% (21/122) of ZA-treated patients. Although the results at any individual skeletal site for any individual variable should be interpreted with caution because of the small population sizes, the present outcomes in our study were comparable to those of other series [21, 22].

Throughout the follow-up period, the risk of SSEs in the denosumab group was nearly 7 times lower than that in the ZA group. However, there was no significant difference in the risk of SSEs within the 2-year period. Some studies claimed that ZA-treated patients had an increased trend in the risk of SSEs compared with denosumab-treated patients [29, 30], which was not consistent with our findings. One reason may be that patients with an average age of 65 years included in the study may influence the rate of SSEs with denosumab treatment; also, there are differences between denosumab and ZA in the distribution of BMD increases in the cortical and trabecular compartments of bone as well as the effect of denosumab on decreasing cortical porosity, which may contribute to increases in the bone strength [30]. Additionally, although denosumab treatment has an established anti-fracture efficacy, there is recent concern about the increased SSE risk following its discontinuation [18]. Simultaneously, the differences in both race and lifestyle between countries can directly influence the results.

The clinical risk factors (including age, premature menopause, personal fracture history, family fracture history, low BMI and chemotherapy history) in the two groups were analysed in the current study. We found that patients with a history of fracture or a family history of fractures had a higher risk of SSEs. The age of menopause, BMI and chemotherapy history had no significant effect on the occurrence of SSEs; the specific reasons for this finding are unclear and may be related to bone.

The limitations of the study include the short follow-up period, small sample size, and lack of follow up evaluation of the changes in the blood calcium and BMD. Nevertheless, the patient population is a strength of the study and suggests that the current findings are applicable to a wide range of postmenopausal women with oestrogen-receptor-positive breast cancer. Additionally, the precision for estimating the percent of SSEs is low, resulting in large confidence intervals. Although the study is powered to detect SSE differences, the analyses in this study failed to exclude subtle fractures that occurred before the endpoint measurement.

## Conclusions

At the time of the cut-off date for this analysis, we conclude, as expected, that denosumab significantly reduced the risk of SSEs compared with ZA in Asian postmenopausal women who had oestrogen-receptor-positive breast cancer regardless of whether they were followed for 2 years or less, which provided further evidence for the superior ability of denosumab to prevent SSEs versus ZA. Further investigation of the long-term utility of denosumab and ZA is necessary.

## Abbreviations

AI: Aromatase inhibitors
ASA: American Society of Anesthesiologists
BMD: Bone mineral density
BMI: Body mass index
HKCRC: Hong Kong Practice-Based Cancer Research Center
RANKL: Receptor Activator of Nuclear Factor Kappa-B Ligand
SDs: Standard deviations
SSEs: Symptomatic skeletal events
TAM: Tamoxifen
ZA: Zoledronic acid
